# 

**Published:** 1872-11-01

**Authors:** 


LIBRARY OF THE SURGEON GENERAL'S OFFICE, U. S. ARMY.

### READER'S CARD.

| AUTHOR. Give Name and Initials. | TITLE OF WORK WANTED. Write title in brief, and give size, place, and date of publication. If a periodical, give year, vol., and page. |
|---|---|
| J H Fitzsimmons | Catarrh of the nose, throat, & Stomach of 8 yr's standing Med Exam Chicago 1872 XIII — 324 326 |

(Name of Reader:) G. B. Price

(Date:) 11 — 22 —, 1897

### RULES FOR READERS.

1. All books must be consulted in the Reading Room, which is open from 9 a. m. until 4 p. m.
2. Fill out this card as above and hand to the Clerk in charge of Reading Room.
3. A separate card is required for each book called for.
4. When desired, books will be set aside a reasonable time for further consultation.
5. Visitors are not permitted to enter the Alcoves nor to take books from the shelves.
6. Readers will please register their names before calling for books.
7. Conversation is not permitted in the Reading Room.

☞ The INDEX CATALOGUE and INDEX MEDICUS furnish complete references to everything in the LIBRARY.

